# The Management of Convalescence

**Published:** 1907-05

**Authors:** 


### THE MANAGEMENT OF CONVALESCENCE.

In convalencence from acute diseases, such as pneumonia, typhoid fever, acute articular rheumatism, etc., we are face to face with the problem of restoring the weakened organism to its normal condition. The blood shows a state of secondary anemia, the nutrition is lowered, the nerve and muscular tone is below par; the appetite but sluggishly answers our urging, and the digestive powers feebly respond to the demands made upon them.

It is at the dawn of convalescence, when the danger of the illness itself has passed, when the desire to live, to get strong, is highest in the patient, that the physician's reputation often hangs in the balance. Having brought the patient through an illness, many physicians are unfortunately content to rest on their laurels, and to let long-suffering "Nature" do the rest. The wise practitioner, however, knows that Nature is grateful for the proper kind of aid in these circumstances,—aid in her efforts to lead a weak organism out of the bondage of illness.

And so, the far-seeing physician will look about in his armamentarium for a drug or a combination of drugs which will restore the blood, the nutrition, the digestion, the assimilation, the appetite, the weight, and the powers of resistance of the sufferer to normal, in the quickest possible time.

Fortunately, nature has provided two chemical elements, iron and manganese, which are as necessary to the system as life itself, and which, when given in the proper amounts and in the proper forms, will carry the patient through convalescence to health. In the delicate state of the digestion of a convalescent it is of the utmost importance that the forms of iron and manganese administered be such as to become absorbed and assimilated with the least disturbance of the gastro-intestinal organs. The old-fashioned inorganic preparations of iron which still figure in the Pharmacopoeias of various countries are totally unsuited for this purpose.

The scientific researches of Hamburger, Bunge, and others, conducted during the past twenty-five years have shown the immeasurable superiority of the organic compounds of iron and maganese. The organic compounds alone have been found to be absorbable in such amounts as to produce the desired action on the blood. Of these compounds the peptonate, which is an organic-chemical combination of iron and manganese with peptone in a solution, known as Pepto-Mangan (Gude) is the most readily absorbed, and therefore the most efficient preparation of iron-manganese known, and as such is used with the greatest benefit in convalescent anemias.

A point which is frequently lost sight of in considering the treatment of anemia, is the importance of manganese as a constituent of normal blood, and as an element ranking only next to iron in its power of building blood corpuscles and increasing the life-bearing hemoglobin of these cells.

Campani, an Italian savant, as early as 1872, demonstrated that manganese is found in the red blood cells, as well as in the serum of normal blood, and the more recent researches of Lecanu and Lheritier show that manganese forms a constant constituent of the hemoglobin molecule. Furthermore, Zaleski (Zeitschr. f. physiol. Chemie, 1904, page 449) showed that manganese enters the molecule of hemoglobin with the same readiness as does iron, and therefore it has the same direct blood-forming power as iron. But, perhaps the most important fact in connection with manganese, is that once having entered the red cell, it attracts iron to the coloring matter of the blood, as the recent investigations of Benedetti have shown (Boll. Scienc. Mediche, Bologna, June, 1905).

A consideration of the above facts will convince any unbiased physician.

physician that the preparation known as Pepto-Mangan (Gude) is made on scientific principles, in accordance with the researches conducted by the foremost physiologists and clinicians within the past quarter of a century. It contains a combination of iron and manganese calculated to secure the highest possible bloodbuilding efficiency without in the least interfering with the digestive functions. On the contrary, Pepto-Mangan is an excellent digestive tonic, it increases the appetite and promotes nutrition. Pepto-Mangan (Gude), therefore offers in convalescence the surest, most agreeable, and most prompt road to perfect health.

